# Identification of circRNA_001846 as putative non–small cell lung cancer biomarker

**DOI:** 10.1080/21655979.2021.1991161

**Published:** 2021-10-25

**Authors:** Fan Yang, Chunlan Ma, Jing Qiu, Xiaoli Feng, Kai Yang

**Affiliations:** a Chengdu Medical College, No. 783, Xindu Avenue, Xindu District, Chengdu (610500), Sichuan. China; b Department of Respiratory and Critical Care Medicine, First Affiliated Hospital of Chengdu Medical College, No. 278, Baoguang Avenue, Xindu District, Chengdu (610500), Sichuan. China; c Key Laboratory of Geriatic Respiratory Diseases of Sichuan Higher Education Institutes, No. 278, Baoguang Avenue, Xindu District, Chengdu (610500), Sichuan. China

**Keywords:** CircRNA, circRNA_001846, prognosis biomarker, NSCLC

## Abstract

CircRNAs play diverse roles in the regulation of oncogenic processes, yet the roles of these circRNAs in non–small cell lung cancer (NSCLC) remain to be fully clarified. Herein, patterns of circRNA expression in NSCLC tissues and paracancerous tissues were assessed, and the relationships between these circRNAs and NSCLC patient clinical findings were assessed. NSCLC tissues were evaluated using a circRNA microarray approach, with Quantitative real‑time polymerase chain reaction (qPCR) qPCR being used to validate candidate circRNA expression levels in NSCLC patients peripheral blood samples. GEO2R was used to analyze the array data. SPSS23.0, GraphPad Prism, and Sigmaplot were utilized for statistical analyses. Overall, 114 circRNAs that were differentially expressed in NSCLC tissue relative to paracancerous tissue levels were identified, of which 77 and 37 were downregulated and upregulated, respectively. CircRNA_001846 were then chosen based on its differential expression score. Consistent with array findings, serum samples from NSCLC patients exhibited circRNA_001846 upregulation relative to those from healthy individuals. The circRNA_001846 was associated with tumor differentiation, lymph node metastasis, and node metastasis stage. Receiver operating characteristic (ROC) curves analyses revealed that levels of circRNA_001846 in the serum were capable of differentiating between individuals diagnosed with NSCLC and controls at a cut off of 3.9496, yielding respective sensitivity and specificity values of 78.2% and 81.1%, with an area under the curve (AUC) value of 0.872. When combined with carcinoembryonic antigen (CEA), this circRNA achieved an improved AUC value of 0.925. CircRNA_001846 may represent a promising diagnostic biomarker for NSCLC.

## Introduction

Non–small cell lung cancer (NSCLC) is among the most common cancers in the world, causing serious harm and mortality in affected patients [1,2]. Approaches to diagnosing and treating this form of malignancy have markedly improved in recent years, such as fungal-derived materials [3], yet patient survival rates remain poor. There is thus a clear need to define a reliable diagnostic biomarker for NSCLC and to explore the value of such biomarkers as targets for therapeutic intervention.

As a recently discovered form of non-coding RNA in the form of a covalently closed loop [4,5], circular RNAs (circRNAs) lack 5ʹ or 3ʹ ends, yet nonetheless harbor sites for microRNA (miRNA) binding [6]. Most circRNAs are derived from exons of protein-coding genes and are produced via a back-splicing process. Owing to their highly stable structural makeup, circRNAs can be readily detected in patient serum samples and in harvested exosomes, making them promising diagnostic targets. Indeed, prior studies have highlighted the value of circRNA-based diagnostic analyses in breast [7,8], colorectal [9,10], liver [11,12], gastric [13,14], and non-small cell lung cancer [15,16]. Functionally, these circRNAs can regulate diverse oncogenic activities such as the migration, proliferation, and survival of tumor cells.

The purpose of this study was to investigate circRNA_001846 as non–small cell lung cancer biomarker. We explored patterns of circRNA_001846 expression in NSCLC patient tumors and paracancerous tissues, and we then evaluated the potential diagnostic utility of an identified NSCLC-related circRNA_001846. Overall, these analyses highlighted the promising value of circRNA_001846 in the diagnostic assessment of potential NSCLC patients.

## Materials and methods

### Sample collection

The the Ethics Committee of First Affiliated Hospital of Chengdu Medical College approved this study, which was conducted in a manner consistent with the declaration of Helsinki. Samples of serum were collected from 206 NSCLC patients and 206 healthy controls following the provision of informed written consent. Blood was collected into a 2 mL sterile RNase-free tube, and RNA was isolated within 30 minutes. Clinicopathological and molecular characteristics were recorded for each patient as appropriate.

### RNA isolation

Trizol (Invitrogen) was used to extract RNA from human samples, after which amirVana Isolation Kit (Ambion, TX, USA) was used based on provided directions. A NanoDrop2000 instrument (Thermo Fisher Scientific) was used to assess RNA quantity and purity, ensuring that OD260/280 ratios were between 1.9 and 2.0. The integrity of isolated RNA was assessed via 1% denatured gel electrophoresis.

### Microarray analysis

A previously published Gene Expression Omnibus (GEO) (https://www.ncbi.nlm.nih.gov/geo/) dataset (GSE158695) was used to assess circRNA expression patterns in NSCLC, with GEO2R (https://www.ncbi.nlm.nih.gov/geo/geo2r/) being used for analysis.

### Quantitative real‑time polymerase chain reaction (qPCR)

A Superscript Reverse Transcription System (Invitrogen) was used to prepare cDNA, after which TB Green qPCR Mastermix (TaKaRa, Japan) and a LightCycler® 480 real-time PCR Platform (Roche) were used to conduct qPCR analyses, which were performed in a total 20 μL reaction volume containing 0.8 μL (10 μM) of forward/reverse primers, 10 μL of TB Green qPCR Mastermix, 2 μL of cDNA, and 6.4 μL double-distilled water. Normalization of gene expression was performed using GAPDH. Primers were prepared by Sangon Biotech (Shanghai) Co. Ltd, circRNA_001846-forward: 5ʹ-CGGCCCTAACAGGGCTCTC-3ʹ, circRNA_001846-reverse: 5ʹ-CCTCTGGCCCTAGTCTCAGAC-3ʹ. The ΔΔCT method was used to assess relative gene expression.

### Enzyme linked immunosorbent assay (ELISA)

Serum CEA levels were measured with third-generation ELISA kits (Can Ag, Canada) based on provided directions. Samples were analyzed in triplicate.

## Statistical analysis

SPSS23.0 (IBM, USA) and GraphPad Prism 7.0 (GraphPad Software, USA) were used to analyze all data, which are given as means ± SD, and were compared via independent samples t-tests or one-way ANOVAs. Receiver operating characteristic (ROC) curves were constructed, and an area under the curve (AUC) value of 0.5 was considered to be indicative of a lack of diagnostic utility. Kaplan-Meier curves were used to compare patient survival outcomes together with log-rank tests, while independent predictors of patient survival were established through univariate and multivariate Cox proportional hazards methods. P < 0.05 was the threshold of significance.

## Results

This study attempted to investigate circRNA_001846 as non–small cell lung cancer biomarker. High levels of serum circRNA_001846 expression were observed in NSCLC. The circRNA_001846 was associated with tumor differentiation, lymph node metastasis, and node metastasis stage. ROC curves analyses revealed that serum circRNA_001846 represents an effective diagnostic biomarker for NSCLC, and the combined evaluation of CEA and serum circRNA_001846 improve an effective diagnostic for NSCLC.

### NSCLC patient circRNA profiling

**Figure 1.**
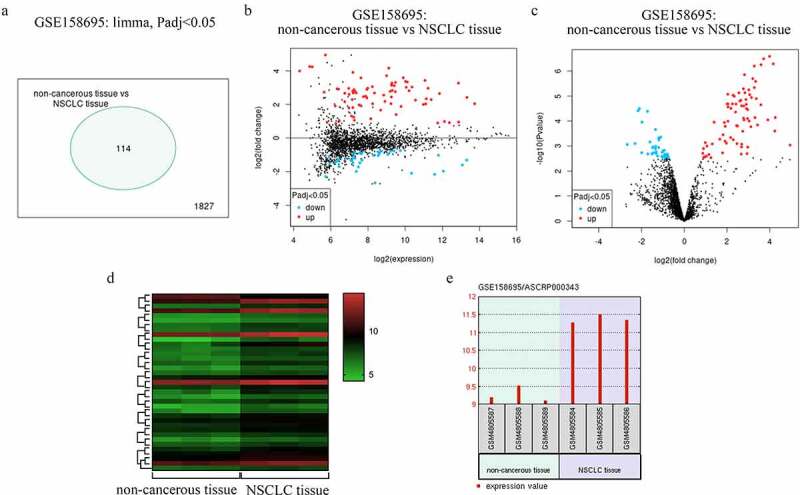
NSCLC patient circRNA profiling results. (a) Differentially expressed circRNAs. (b) Mean-difference plot for the GSE158695 dataset. (c) Differentially expressed genes in the GSE158695 dataset were presented using a Volcano plot. (d) Genes upregulated in the GSE158695 dataset were subjected to clustering analysis. (e) NSCLC samples exhibited circRNA_001846 is upregulation as compared to paracancerous tissue samples

By analyzing a previously published GEO dataset (GSE158695), we identified 114 circRNAs that were differentially expressed in NSCLC patient tumor samples (Figure 1(a-c)). These circRNAs were able to effectively differentiate between NSCLC samples and paracancerous tissues (Figure 1(d)), and of these, circRNA_001846 was the most highly upregulated in NSCLC relative to normal tissue levels (Figure 1(e)). It was thus selected as a target for subsequent study.

### Baseline patient characteristics

To evaluate circRNA_001846 in greater detail, we utilized samples from NSCLC patients and healthy controls (n = 206 each, Table 1). Of these NSCLC patients, 111 and 95 were respectively diagnosed with adenocarcinomas and squamous cell carcinoma. Moreover, 107 patients exhibited lymph node metastases, and 105 and 101 patients, respectively, exhibited good to moderate differentiation and poor differentiation. Based on the TNM staging system, 109 patients had stage I/II disease, while 97 had stage III/IV disease. Patient clinical characteristics are compiled in Table 2.

**Table 1.** NSCLC and control patient clinicopathological characteristics

Characteristic	NSCLC patients	Healthy controls	P
Age			0.152
>60	121 (58.7%)	113 (54.9%)	
≦60	85 (41.3%)	93 (45.1%)	
Gender			0.419
Male	112 (54.4%)	106 (51.5%)	
Female	94 (45.6%)	100 (48.5%)	
Body mass index, kg/m[^2]^	23.12 ± 4.02	25.29 ± 5.32	0.361
Smoking (%)	153 (74.3%)	83 (40.3%)	0.023
Drinking (%)	125 (60.7%)	116 (56.3%)	0.518
Type of NSCLC (%)			
Adenocarcinoma	111 (53.9%)		
Squamous cell carcinoma	95 (46.1%)		
Lymph node metastasis (%)			
Positive	99 (48.1%)		
Negative	107 (51.9%)		
TNM stage (%)			
I + II	109 (52.9%)		
III + IV	97 (47.1%)		
Tumor differentiation (%)			
Well + moderate	105 (51.0%		
Poor	101 (49.0%)

**Table 2.** Association between levels of circRNA_001846 expression and clinicopathological characteristics

Clinical features	Case	circRNA_001846 expression		
		Low	High	P-value
Age				0.158
>60	121	63	58	
≦60	85	40	45	
Gender				0.128
Male	112	54	58	
Female	94	49	45	
Histological type				0.097
Adenocarcinoma	111	51	60	
Squamous cell carcinoma	95	52	43	
Smoking status				0.209
Ever	153	72	81	
Never	53	31	22	
Lymph node metastasis				0.002
Positive	99	29	70	
Negative	107	74	33	
Tumor differentiation				0.001
Well + moderate	105	81	24	
Poor	101	22	79	
TNM stage				0.001
I + II	109	29	80	
III + IV	97	74	23	

### NSCLC patients exhibit increased serum circRNA_001846 expression

**Figure 2.**
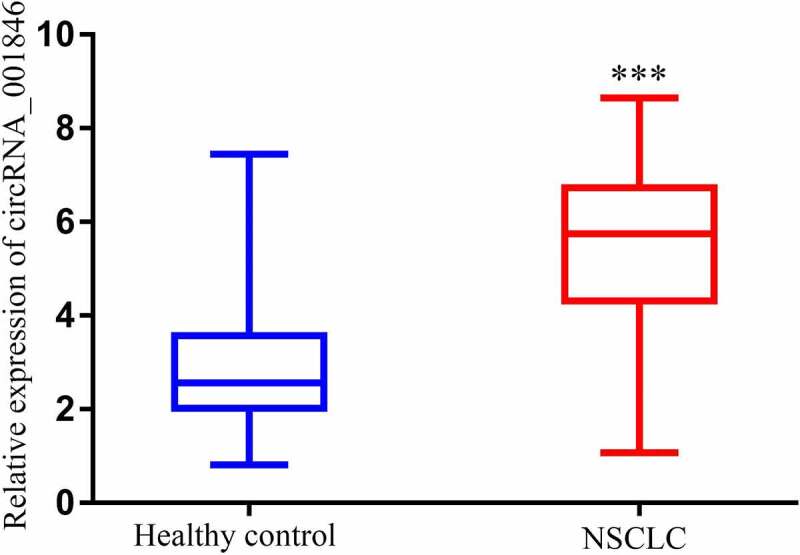
Serum circRNA_001846 in NSCLC patients. Levels of serum circRNA_001846 were higher in patients with NSCLC relative to healthy individuals. ***p < 0.001

A qPCR approach revealed that NSCLC patients exhibited higher serum circRNA_001846 levels as compared to healthy study participants (Figure 2).

### Serum circRNA_001846 levels are related to NSCLC patient clinicopathological characteristics

**Figure 3.**
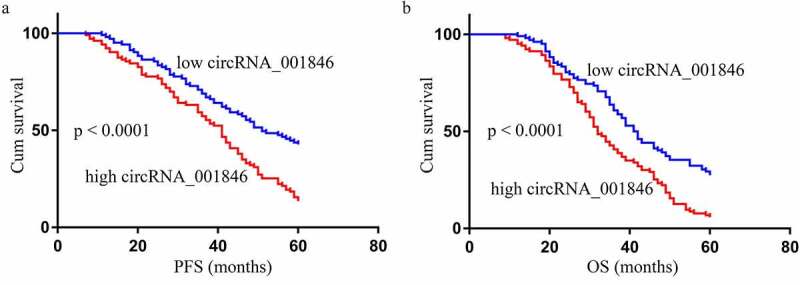
The relationship between levels of circRNA_001846 in NSCLC patient serum and associated clinicopathological characteristics. PFS (a) and OS (b) were compared between patients expressing low and high levels of circRNA_001846 via log-rank tests

Next, NSCLC patients in our study cohort were stratified into those expressing low and high levels of serum circRNA_001846 (n = 103 each) based upon the median expression of this circRNA among these patients. Subsequent analyses of the clinical characteristics of patients in these two groups revealed high circRNA_001846 levels to be associated with lymph node metastasis (P = 0.002), poor tumor differentiation (P = 0.001), and advanced tumor node metastasis (TNM) stage (P = 0.001) (Table 2). These levels were unrelated to patient age (P = 0.158), sex (P = 0.128), histological type (P = 0.097), or smoking status (P = 0.209). Moreover, higher serum levels of this circRNA were linked to significantly reduced progression-free survival (PFS, Figure 3(a)) and overall survival (OS, Figure 3(b)) among NSCLC patients as compared to those of patients with low serum circRNA_001846 levels. Consistent with these results, serum circRNA_001846 levels were correlated with PFS (P = 0.002, Table 3) and OS (P = 0.005, Table 4) in univariate analyses, as and were also associated with significant reductions in patient PFS (P = 0.001, Table 3) and OS (P = 0.008, Table 4) in multivariate analyses.

**Table 3.** Univariate and multivariate Cox analysis of factors related to NSCLC patient PFS

Characteristics	Univariate		Multivariate	
	HR (95% CI)	P	HR (95% CI)	P
Gender (male vs female)	1.065 (0.562–1.835)	0.523	0.854 (0.523–1.254)	0.821
Age (<60 vs ≥60)	0.751 (0.645–1.052	0.251	0.652 (0.352–1.025	0.171
Histological type (SCC vs ADC)	0.625 (0.251–1.228)	0.352	1.121 (0.856–1.562)	0.208
Differentiation (well-moderate vs poor)	0.518 (0.412–0.632)	0.041	0.854 (0.695–1.065)	0.037
TNM stage (I–II vs III–IV)	1.025 (0.953–1.125)	0.026	1.254 (1.025–2.052)	0.024
Lymph node metastasis (negative vs positive)	0.554 (0.415–0.751)	0.034	1.019 (0.863–1.365)	0.028
circRNA_001846 (low vs high)	2.121 (1.254–3.842)	0.002	2.952 (1.562–5.657)	0.001

**Table 4.** Univariate and multivariate Cox analysis of factors related to NSCLC patient OS

Characteristics	Univariate		Multivariate	
	HR (95% CI)	P	HR (95% CI)	P
Gender (male vs female)	0.914 (0.415–1.759)	0.842	0.652 (0.254–1.319)	0.325
Age (<60 vs ≥60)	0.925 (0.325–2.054)	0.711	1.125 (0.522–2.412)	0.319
Histological type (SCC vs ADC)	0.524 (0.194–1.754)	0.351	1.852 (0.612–1.519)	0.152
Differentiation (well-moderate vs poor)	1.254 (0.913–1.952)	0.033	1.025 (0.524–1.512)	0.041
TNM stage (I–II vs III–IV)	1.161 (0.722–1.543)	0.029	1.244 (1.037–1.612)	0.032
Lymph node metastasis (negative vs positive)	1.954 (1.354–2.854)	0.038	1.007 (0.996–1.751)	0.036
circRNA_001846 (low vs high)	2.364 (1.342–4.853)	0.005	2.241 (1.119–3.997)	0.008

### Assessment of the diagnostic utility of serum CEA in NSCLC

**Figure 4.**
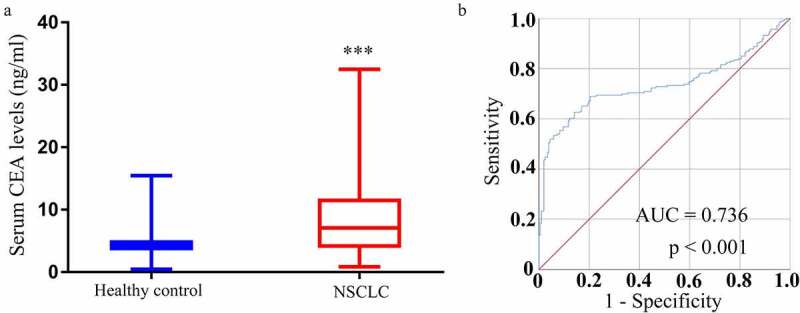
CEA expression and diagnostic utility in NSCLC. (a) Levels of CEA in serum samples were higher in individuals with NSCLC as compared to controls. (b) Serum CEA levels were capable of differentiating between individuals with NSCLC and controls in an ROC curve analysis

CEA is perhaps the best-studied serum biomarker used to detect NSCLC at an early stage. The mean serum CEA levels among NSCLC patients and controls in this study were 14.82 ± 17.24 ng/mL and 3.37 ± 2.74 ng/mL (P < 0.0001) (Figure 4(a)). ROC curves revealed that these serum CEA levels were able to effectively differentiate between NSCLC patients and controls with an area under the curve (AUC) of 0.736 (95% confidence interval [CI]: 0.685–0.787) at a cutoff level of 5.731 ng/mL (Figure 4(b)), yielding respective diagnostic sensitivity and specificity values of 62.6% and 85.9%.

### Serum circRNA_001846 represents an effective diagnostic biomarker for NSCLC

**Figure 5.**
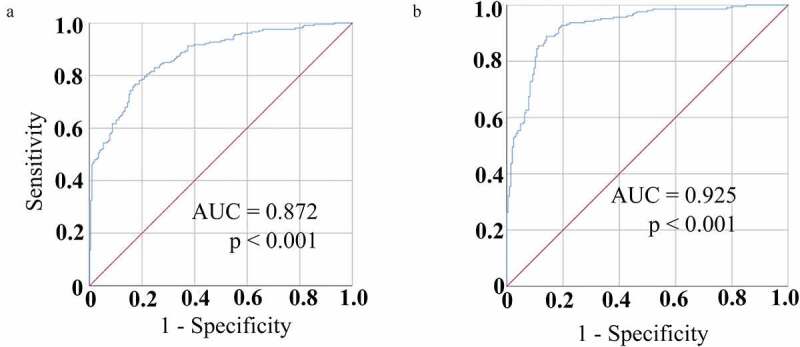
Serum circRNA_001846 diagnostic utility in NSCLC. (a) Serum circRNA_001846 levels enabled effective differentiation between individuals with NSCLC and controls in an ROC curve analysis. (b) An ROC curve was generated for a combination of both CEA and circRNA_001846 as an approach to differentiating NSCLC patients from healthy controls

A similar analysis of the diagnostic value of serum circRNA_001846 levels in the evaluation of NSCLC patients was next performed, revealing that these levels could readily differentiate between NSCLC patients and controls with an AUC of 0.872 (95% CI: 0.839–0.906) at an optimal cutoff value of 3.9496 (Figure 5(a)), yielding respective sensitivity and specificity values of 78.2% and 81.1%. As such, these results suggested that circRNA_001846 levels may be more sensitive than those of CEA when evaluating patients for NSCLC.

To expand upon these findings, we assessed the combined diagnostic value of serum CEA and circRNA_001846 in NSCLC patients. This combined analysis yielded an AUC of 0.925 (95% CI: 0.899–0.950), with respective sensitivity and specificity values of 88.8% and 85.9% at an optimal cutoff of 4.9467, with predictive probabilities above this cutoff level being considered positive for NSCLC.

## Discussion

NSCLC is among the most prevalent and deadly tumors in the world, yet patients are often only diagnosed when their disease has already reached an advanced stage at which time they exhibit relatively poor 5-year survival outcomes [17]. The early diagnosis and treatment of these patients is thus vital to ensure their improved treatment outcomes. Currently, approaches used to screen for NSCLC tumors include computed tomography, magnetic resonance imaging, positron emission tomography, and sputum cytology. However, these approaches can be invasive, expensive, and can expose patients to radioactivity [18–20]. In addition, circulating tumor biomarkers may manifest in the periphery at an earlier time point than imaging can detect lung tumors, offering an opportunity to detect otherwise asymptomatic early-stage disease [21]. Evaluating peripheral blood biomarkers thus represents an attractive, noninvasive, inexpensive means of diagnosing NSCLC.

The identification of circRNAs as stable and highly abundant non-coding RNAs with direct oncogenic relevance has been a focus of intensive research interest in recent years as a means of gauging cancer patient prognosis and guiding diagnostic efforts [22–24]. These circRNAs remain stable in patient plasma and serum, and are resistant to the effects of pH shifts or freeze-thawing [25,26]. As such, they are ideal targets for the blood testing-based evaluation of patients in a minimally invasive manner for a range of cancers. For example, Pan et al. found that levels of has_circ_0004771 in patient serum were predictive of colorectal cancer status [27], while serum has_Circ_0141633 has shown promise as a gastric cancer biomarker [28], and Sun et al. found circRNA_001846 to be a promising gastric carcinoma-related biomarker [29]. Functionally in cervical cancer cells, the upregulation of circRNA_001846 led to shifts in miR-1296 activity and consequent cyclin-dependent kinases 2 (CDK2) upregulation, ultimately driving proliferative activity [30]. No prior studies have reported on the diagnostic utility of circRNA_001846 in NSCLC.

Here, we explored the potential diagnostic utility of circulating circRNA_001846 in the context of NSCLC. We found that the upregulation of this circRNA in patient serum was associated with lymph node metastasis, tumor differentiation, and TNM staging, suggesting a potential functional role for circRNA_001846 as a regulator of NSCLC onset or progression. Moreover, higher serum circRNA_001846 levels were linked to worse NSCLC patient OS and PFS, suggesting that it may represent a valuable prognostic biomarker capable of guiding patient management efforts. The diagnostic value of serum circRNA_001846 was further evaluated using ROC curves, revealing that it was able to differentiate between NSCLC patients and healthy controls with excellent sensitivity and specificity that were superior to those of CEA. Moreover, the combined evaluation of CEA levels and serum circRNA_001846 expression achieved 90% specificity in the diagnostic evaluation of NSCLC patients at a cutoff value of 0.678. Such combined biomarker-based analytical approaches may thus represent a potent means of reliably identifying NSCLC patients in the clinical setting.

## Conclusion

In summary, circRNA_001846 upregulation was evident in individuals with NSCLC, and such upregulation may be linked to disease incidence. The serum levels of this circRNA may offer value as a diagnostic biomarker for early-stage NSCLC. By combining this biomarker with other standard clinical markers, it may be possible to achieve superior diagnostic reliability. Despite the limitations of this analysis, our results nonetheless highlight directions for future research evaluating the clinical utility of circRNA_001846 when screening for patients with early-stage NSCLC.

## Highlights

1. NSCLC patients exhibit increased serum circRNA_001846
2. Serum circRNA_001846 are related to NSCLC patient clinicopathological characteristics
3. Serum circRNA_001846 represents an effective diagnostic biomarker for NSCLC

The combined evaluation of CEA and serum circRNA_001846 improve an effective diagnostic for NSCLC
